# The contribution of nutrient metal acquisition and metabolism to *Acinetobacter baumannii* survival within the host

**DOI:** 10.3389/fcimb.2013.00095

**Published:** 2013-12-12

**Authors:** Brittany L. Mortensen, Eric P. Skaar

**Affiliations:** Department of Pathology, Microbiology, and Immunology, Vanderbilt University School of MedicineNashville, TN, USA

**Keywords:** *Acinetobacter*, pathogenesis, iron, zinc, adherence, persistence

## Abstract

*Acinetobacter baumannii* is a significant contributor to intensive care unit (ICU) mortality causing numerous types of infection in this susceptible ICU population, most notably ventilator-associated pneumonia. The substantial disease burden attributed to *A. baumannii* and the rapid acquisition of antibiotic resistance make this bacterium a serious health care threat. *A. baumannii* is equipped to tolerate the hostile host environment through modification of its metabolism and nutritional needs. Among these adaptations is the evolution of mechanisms to acquire nutrient metals that are sequestered by the host as a defense against infection. Although all bacteria require nutrient metals, there is diversity in the particular metal needs among species and within varying tissue types and bacterial lifecycles. *A. baumannii* is well-equipped with the metal homeostatic systems required for the colonization of a diverse array of tissues. Specifically, iron and zinc homeostasis is important for *A. baumannii* interactions with biotic surfaces and for growth within vertebrates. This review discusses what is currently known regarding the interaction of *A. baumannii* with vertebrate cells with a particular emphasis on the contributions of metal homeostasis systems. Overall, published research supports the utility of exploiting these systems as targets for the development of much-needed antimicrobials against this emerging infectious threat.

## Introduction

The genus *Acinetobacter* is comprised of a number of Gram-negative species that are ubiquitous in the environment. Notably, some of the species within this genus have emerged as opportunistic human pathogens. *A. baumannii* is the most notorious among *Acinetobacter* species for causing a wide range of hospital-acquired infections. This organism is particularly problematic in intensive care units (ICUs) and can cause urinary tract infections, wound and burn infections, sepsis, ventilator-associated pneumonia, meningitis, and osteomyelitis (Davis et al., 2005; Peleg et al., 2008; Doyle et al., 2011). There have been numerous reports of *A. baumannii* wound infections in military personnel returning from Iraq and Afghanistan (Whitman, 2007; Calhoun et al., 2008; Sebeny et al., 2008). Moreover, *A. baumannii* causes community-acquired infections, predominately in Asia and tropic Australia (Falagas et al., 2007). *A. baumannii* infection is facilitated through biofilm formation on indwelling medical devices and other hospital surfaces and the ability to withstand desiccation and disinfection (Vidal et al., 1996; Wendt et al., 1997; Neely, 2000; Tomaras et al., 2003; Kawamura-Sato et al., 2010; Pour et al., 2011). In addition to the wide repertoire of diseases caused by *A. baumannii*, this organism has acquired antibiotic resistance at alarming rates. In fact, multi-drug resistance has become commonplace, and pan-drug resistant strains are increasingly identified. This is reflected in part by the fact that *A. baumannii* displays significant genomic plasticity and intraspecies heterogeneity (Fournier et al., 2006; Vallenet et al., 2008; Adams et al., 2010; Sahl et al., 2011, 2013). These facts highlight the necessity to develop new antimicrobials against *A. baumannii.*

*A. baumannii* can adapt to the hostile host environment through modification of its metabolism and nutritional needs. Like all organisms, *A. baumannii* requires nutrient metals to survive. Generally these essential metals include iron, zinc, manganese, copper, magnesium, and nickel, which serve as co-factors for a variety of fundamental cellular processes. Furthermore, *A. baumannii* displays diverse tissue tropism and has evolved mechanisms to acquire nutrient metals in various niches within the host. Although the lifecycle of *A. baumannii* in these tissues is not well-defined, several bacterial factors have been identified that are required for host cell interactions during infection. This review discusses *A. baumannii* interactions with host cells with a particular emphasis on the importance of nutrient metal homeostasis to the pathogenesis of *A. baumannii* infection.

## Nutritional immunity

In order to adapt to different host niches, *A. baumannii* must possess metabolic flexibility and employ specialized systems for nutrient acquisition and homeostasis. Humans maintain extracellular free metals at low levels through several mechanisms including intracellular localization and the expression of metal-binding proteins. This provides protection against invading pathogens that must acquire metals from their hosts. Therefore, metal limitation is considered a host defense mechanism and has been termed nutritional immunity (Weinberg, 1975). Research into nutritional immunity has primarily focused on iron. Most iron in vertebrates is stored within heme, the oxygen-carrying cofactor of the protein hemoglobin, which is stored within erythrocytes. In addition to hemoglobin, the host has other iron transport and storage proteins that reduce iron availability in the vertebrate host, including myoglobin, transferrin, lactoferrin, hemopexin, and ferritin.

Vertebrates also limit non-iron metals to defend against infection. Calprotectin (CP) is a heterodimer of the two S100 proteins, S100A8 and S100A9, and exhibits high affinity binding for zinc and manganese. Importantly, CP displays antimicrobial activity against several pathogens, including *A. baumannii*, via its metal-sequestering properties (Corbin et al., 2008; Urban et al., 2009; McCormick et al., 2010; Bianchi et al., 2011; Damo et al., 2013). CP is frequently identified at sites of inflammation, including in the lungs during *A. baumannii* pneumonia where CP expression tracks with the progression and resolution of infection (Hood et al., 2012; Moore et al., 2013). In addition to CP, other S100 proteins have antimicrobial activity and the ability to sequester metals including S100A7 (psoriacin), S100A12, and S100A15 (Glaser et al., 2005; Buchau et al., 2007; Lee and Eckert, 2007; Michalek et al., 2009; Pietzsch and Hoppmann, 2009). Other than CP, these extracellular metal-binding proteins have yet to be investigated for their role in protection against *A. baumannii* infections.

Finally, in order to limit metal availability to invading pathogens, vertebrate cells efflux iron and manganese out of the phagosomal compartment into the cytoplasm via NRAMP1 (Jabado et al., 2000; Peracino et al., 2006; Cellier et al., 2007). Phagosomal zinc levels are decreased via ZIP8-mediated transport into the cytoplasm (Begum et al., 2002; Aydemir et al., 2009). Cytoplasmic zinc levels are then reduced via cellular extrusion by ZnT transporters (Kitamura et al., 2006; Murakami and Hirano, 2008). Vertebrate systems such as these are important to protect against intracellular bacterial growth but have yet to be studied in the context of *A. baumannii* infection.

## Iron and *A. baumannii*

In response to low iron availability within the host, both Gram-negative and Gram-positive bacteria employ the iron-dependent repressor ferric uptake regulator (Fur), which regulates gene expression through binding of a conserved Fur box DNA sequence upstream of target genes. *A. baumannii* Fur has been identified and is expressed within the *A. baumannii* strain BM2580 and has 63% identity to the *Escherichia coli* Fur (Daniel et al., 1999). Furthermore, Fur boxes have been identified within the genomes of *A. baumannii* strains ATCC 17978 and ATCC 19606^T^ (Mihara et al., 2004; Eijkelkamp et al., 2011a). *A. baumannii* responds to iron starvation by modifying gene expression for many predicted iron-related genes, as well as for genes involved in various processes such as respiration, biofilm formation, and motility, highlighting the importance of iron levels to *A. baumannii* virulence (Eijkelkamp et al., 2011a; Nwugo et al., 2011). This observation is consistent with the function of Fur in other bacterial species where Fur has been reported to regulate similar processes including genes required for virulence (Troxell and Hassan, 2013). Although not yet demonstrated for *A. baumannii*, in several organisms Zur is required for pathogenesis in *in vivo* models of infection, including *Staphylococcus aureus, Vibrio cholera, Salmonella enterica* serovar Typhimurium, and *Helicobacter pylori* (Bury-Mone et al., 2004; Mey et al., 2005; Gancz et al., 2006; Velayudhan et al., 2007; Curtiss et al., 2009; Torres et al., 2010; Troxell et al., 2011). Numerous studies have investigated the response of various *A. baumannii* isolates to iron-limiting conditions and demonstrated changes in outer membrane protein composition and/or secretion of iron-chelating siderophores, which are discussed further below (Echenique et al., 1992; Actis et al., 1993; Yamamoto et al., 1994; Goel et al., 1998; Daniel et al., 1999; Dorsey et al., 2003a). Interestingly, among *A. baumannii* strains, there is significant diversity in the numbers and types of these iron uptake and utilization systems that are expressed (Yamamoto et al., 1994; Dorsey et al., 2003a; Zimbler et al., 2009; Antunes et al., 2011).

There are several generally conserved Gram-negative mechanisms for capturing iron or iron-protein complexes through specific receptors that are utilized by *A. baumannii*. Many bacterial pathogens lyse erythrocytes in order to initiate the coordinated process of freeing heme to extract the bound iron as a source of this essential metal. *A. baumannii* encodes a phospholipase C and other hemolysin-related genes that can lyse horse erythrocytes (Vallenet et al., 2008; Camarena et al., 2010; Antunes et al., 2011). Following erythrocyte lysis, bacteria can then capture iron-bound heme via specialized heme uptake systems (Runyen-Janecky, 2013). Among *A. baumannii* sequenced strains, there are at least two identified gene clusters encoding putative heme uptake systems, one of which is highly conserved among the sequenced strains (Zimbler et al., 2009; Antunes et al., 2011). The process of iron extraction from heme has not yet been described for *A. baumannii.*

Bacteria can also acquire iron from other host iron-binding proteins through the use of secreted siderophores. *A. baumannii* encodes several iron-scavenging siderophores, and at least five gene clusters for siderophore synthesis and transport have been discovered among the sequenced strains. The process of encoding more than one siderophore is not unique to *A. baumannii*, as several other organisms encode more than one siderophore including *E. coli, Pseudomonas aeruginosa, Yersinia pestis, Mycobacterium tuberculosis*, and *S. aureus* (Hammer and Skaar, 2011; Chaturvedi et al., 2012; Rakin et al., 2012; Li et al., 2013; Saha et al., 2013). The first identified *A. baumannii* siderophore cluster, comprised of 10 ORFs, was discovered in strain 8399 and several other clinical isolates from Oregon (Echenique et al., 1992; Dorsey et al., 2003b). This 8399 cluster is responsible for the production of a catechol siderophore that chelates oxidized ferric iron from transferrin and restores *A. baumannii* growth when iron is limiting. Other bacteria that produce catechol siderophores include *Bacillus subtilis, Streptomyces, S. aureus, E. coli, V. cholera, Y. pestis*, and *P. aeruginosa* (May et al., 2001; Chu et al., 2010; Saha et al., 2013). Specifically, the genes in the cluster encode proteins with similarity to *B. subtilis* DhbB, DhbE, DhbC, DhbA, DhbF, that are related to the Ent proteins required for enterobactin synthesis in *E. coli* (Rowland et al., 1996; Dorsey et al., 2003b). This cluster also encodes proteins with similarity to the *Y. pestis* putative enterobactin biosynthesis protein EntD, a *Y. pestis* iron-regulated efflux protein P114, *E. coli* siderophore efflux protein EntS, and the *E. coli* enterobactin degradation protein Fes (Armstrong et al., 1989; Coderre and Earhart, 1989; Brickman and McIntosh, 1992; Parkhill et al., 2001; Furrer et al., 2002). Finally, an ORF encoding a 73 kDa outer membrane protein OM73 was identified in the cluster and has homology to the *E. coli* CirA colicin receptor protein (Nau and Konisky, 1989). OM73 is surface-exposed, Fur- and iron-regulated, and contains a TonB box, which is consistent with described siderophore receptors (Dorsey et al., 2003b).

The most-studied *A. baumannii* siderophore gene cluster encodes the siderophore acinetobactin, which is found in numerous clinical isolates and in all sequenced genomes except *A. baumannii* SDF (Yamamoto et al., 1994; Dorsey et al., 2004; Mihara et al., 2004). The structure of acinetobactin is composed of equimolar amounts of 2,3-dihydrobenzoic acid (DHBA), threonine, and N-hydroxyhistamine (Yamamoto et al., 1994). Hydroxamate or mixed-type siderophores are also found in a wide array of bacterial species (Chu et al., 2010; Saha et al., 2013). In fact, acinetobactin is structurally related to the *Vibrio anguillarum* siderophore anguibactin, and acinetobactin only differs by the presence of an oxazoline ring instead of thiazoline ring (Yamamoto et al., 1994). Acinetobactin can functionally replace anguibactin for iron acquisition in a *V. anguillarum* mutant that cannot produce anguibactin (Dorsey et al., 2004). In addition to being regulated by iron levels, the acinetobactin gene cluster is regulated by Fur, and Fur boxes have been identified within the cluster (Mihara et al., 2004). Homology analyses identified three putative systems encoded within the acinetobactin cluster: *basABCDEFGHIJ* for *A. baumannii* acinetobactin synthesis, *bauABCDEF* for *A. baumannii* acinetobactin utilization, and *barAB* for *A. baumannii* acinetobactin release (Mihara et al., 2004). Of note, *basF* and *basJ* genes in the Fur-regulated cluster are homologs of the *E. coli entC* and *entB*, which are required for the production of DHBA; however, uniquely, *A. baumannii* encodes the *entA* homolog elsewhere in the genome (Mihara et al., 2004; Penwell et al., 2012). Although *entA* is found outside of the acinetobactin cluster in all strains investigated, the genomic context surrounding the *entA* gene differs by strain, highlighting the variability, and plasticity of the *A. baumannii* genome. EntA in *A. baumannii* ATCC 19606^T^ is required for production of DHBA and for iron acquisition (Penwell et al., 2012). Analysis of a *basD* mutant demonstrated that no acinetobactin is produced and these mutants have a growth defect in iron-limiting conditions, consistent with the predicted function in acinetobactin synthesis (Dorsey et al., 2004; Mihara et al., 2004). The putative function of the *bauABCDEF* system in acinetobactin uptake was demonstrated through the use of *bauA* and *bauD* mutants, which can produce acinetobactin but cannot grow in conditions where ferric iron or transferrin-bound iron are the sole iron source (Dorsey et al., 2004; Mihara et al., 2004). Moreover, BauA has homology to FatA, which is the ferric-anguibactin outer membrane receptor belonging to the family of TonB-dependent receptors (described below), and BauA can be recognized by anti-FatA antibodies (Dorsey et al., 2004).

The third described siderophore biosynthesis cluster encodes a system that produces six siderophores termed fimsbactins A-F found in *A. baumannii* ATCC 17978 and ADP1 (Proschak et al., 2013). A fourth siderophore gene cluster (ACICU1672-1683) is conserved among the sequenced strains, except SDF; however, this cluster has yet to be empirically studied (Antunes et al., 2011; Eijkelkamp et al., 2011a). The final identified siderophore gene cluster (ABAYE1888-1889) contains two genes encoding an isochorismatase and a 2,3-dihydro-2,3-hydroxybenzoate dehydrogenase (Eijkelkamp et al., 2011a). These enzymes produce 2,3-dihydroxybenzoate, which is an iron-binding molecule but also a precursor for more complex siderophores. This fifth cluster is not conserved among all strains and remains to be experimentally assessed (Eijkelkamp et al., 2011a). Elucidation of the functions of the other siderophore gene clusters will provide insight into why *A. baumannii* exhibits such significant diversity in its iron acquisition systems.

The ability of a siderophore-iron complex to be transported into Gram-negative bacterial cells is reliant on an outer membrane TonB-dependent receptor, named such for its dependence on a TonB/ExbB/ExbD energy-transducing system. Many bacteria encode multiple TonB proteins required under different conditions and/or for varying purposes extending beyond siderophore transport to include the transport of heme, maltose, vitamin B_12_, and nickel (Lewis et al., 1997; Chimento et al., 2003; Neugebauer et al., 2005; Schauer et al., 2007; Krewulak and Vogel, 2011). In fact, *A. baumannii* strains contain from 8 to 22 predicted TonB-dependent receptors in the genome. The TonB-dependent receptor BauA is involved in acinetobactin transport, but the functions of the remaining TonB-dependent receptors are not known. Three TonB systems are conserved in all sequenced *A. baumannii* genomes, and recent work demonstrates that the genes encoding each of these systems were likely horizontally acquired from distinct sources (Zimbler et al., 2013). These three systems in *A. baumannii* ATCC 19606^T^ are *tonB_1_/exbB_1_/exbD_1.1_/exbD_1.2_, tonB_2_, and tonB_3_/exbB_3_/exbD_3_.* The expression and function of each *A. baumannii* TonB system is variable. Of these systems, only *tonB*_*3*_ is up-regulated under iron-limiting conditions, indicating a link to iron homeostasis. Additionally, only TonB_2_ and TonB_3_ can functionally complement the iron-limited growth defect of an *E. coli tonB* mutant. Interestingly, both ExbB_1_/ExbD_1.1_/ExbD_1.2_ and ExbB_3_/ExbD_3_ are required for complementation of *E. coli exbBD tolQR* mutant growth. Within *A. baumannii, tonB*_*1*_, *tonB*_*2*_, and *tonB*_*1*_ *tonB*_*2*_ mutants are deficient for growth in iron-starved conditions as compared to wild-type and are less efficient at acinetobactin and iron transport. A *tonB*_*3*_ mutant has not been created, which may indicate that this gene is required for growth (Zimbler et al., 2013).

In certain ecological niches, including the intracellular compartment, bacteria may be exposed to reduced ferrous iron as an iron source. Therefore, many bacteria encode ferrous iron uptake systems, most notably FeoAB transporters, which are required for iron acquisition and pathogenesis of numerous bacteria including *E. coli, Shigella flexneri, H. pylori*, and *Legionella pneumophila* (Stojiljkovic et al., 1993; Velayudhan et al., 2000; Robey and Cianciotto, 2002; Runyen-Janecky et al., 2003; Cartron et al., 2006). *A. baumannii* encodes putative ferrous iron import systems, FeoAB with its regulator FeoC, and at least one FeoB has been identified in all sequenced strains along with a FeoA and FeoC (Antunes et al., 2011). Finally, once in the bacterial cell, iron must be distributed to the cytosolic iron pool or incorporated into proteins requiring iron cofactors. One common class of iron metallocenter found in a variety of proteins are iron-sulfur clusters, whose formation is dependent on a metallocenter assembly scaffold of three main types: NIF, ISC, and SUF (Bandyopadhyay et al., 2008a). The Nfu proteins are a class of scaffold proteins outside of the three main iron-sulfur cluster types and have been described in eukaryotes, *E. coli*, and *Azotobacter vinelandii* (Angelini et al., 2008; Bandyopadhyay et al., 2008a,b; Py et al., 2012). *A. baumannii* NfuA is a cytoplasmic protein that binds iron and is predicted to function in iron-sulfur (Fe-S) cluster formation (Zimbler et al., 2012). Consistent with this function, NfuA is required for growth in low iron and in conditions of oxidative stress (Zimbler et al., 2012).

**Figure 1:**
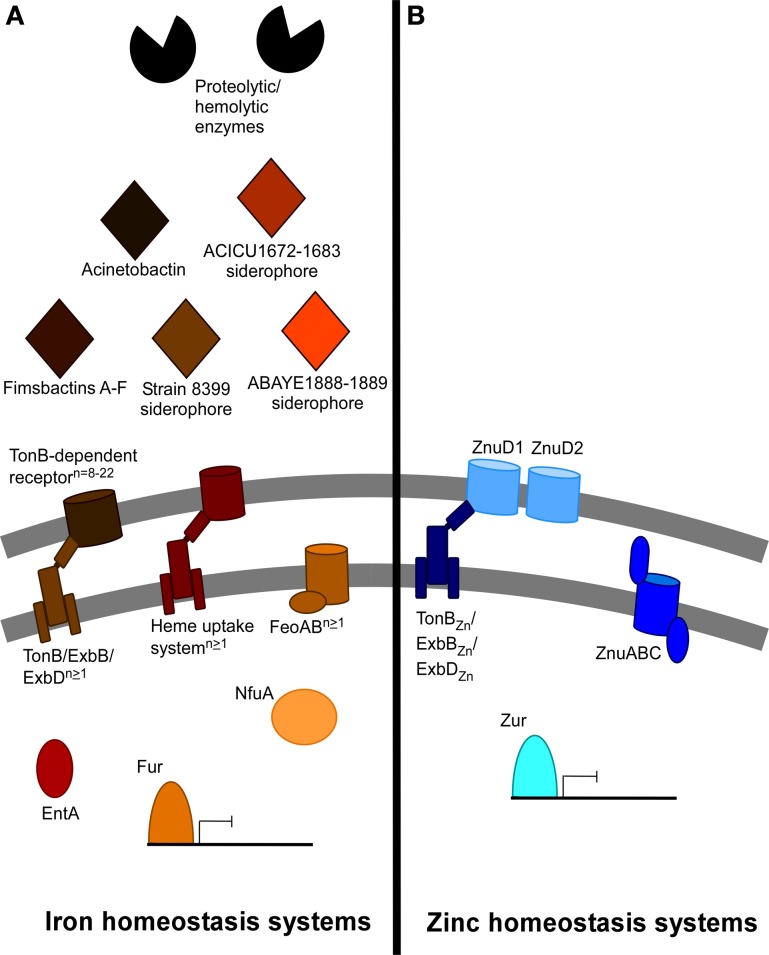
**Summary of predicted and described iron and non-iron metal acquisition systems in *A. baumannii***. The iron and non-iron systems encoded within the *A. baumannii* genome vary dramatically between different strains. **(A)** This panel depicts the iron acquisition and utilization systems identified or described among the different *A. baumannii* strains. Secretion of enzymes such as phospholipase C and others may contribute to hemolysis and hemoglobin release from red blood cells. At least five clusters of genes for siderophore synthesis and secretion are dispersed among different strains. These include acinetobactin, fimsbactins A-F, the strain 8399 (*om73-entD*) siderophore, as well as siderophores produced by the ACICU1672-1683 and the ABAYE1888-1899 clusters. EntA is required for iron acquisition due to its involvement in the biosynthesis of the acinetobactin precursor 2,3-dihydroxybenzoic acid. Siderophores are recognized by TonB-dependent receptors, of which 8–22 have been discovered among the sequenced strains. At least one of multiple encoded TonB/ExbB/ExbD systems generates and provides energy to the TonB-dependent receptors, and at least one FeoAB ferrous iron transport system has been identified in all sequenced strains. Finally, 1–2 heme uptake systems have been identified among *A. baumannii* strains. NfuA is a Fe-S cluster protein required for iron utilization in the cytoplasm. Regulation of these numerous systems likely depends on the conserved Fur repressor. **(B)** This panel depicts the zinc acquisition and metabolism systems described in *A. baumannii* ATCC 17978. Outer membrane transport of zinc may occur via two zinc-regulated TonB-dependent receptors, energized by a zinc-regulated TonB/ExbB/ExbD system. Inner membrane transport is mediated by the ZnuABC transporter. Zur is a conserved repressor that controls the expression of *znuABC* and likely the other identified zinc systems.

At least several of the above iron acquisition and metabolism systems are required for *A. baumannii* infection. Acinetobactin synthesis and uptake proteins BasD and BauA, respectively, are required for *A. baumannii* virulence in a *Galleria mellonella* larvae infection model and in a mouse model of systemic infection (Gaddy et al., 2012). Consistent with this, the DHBA production protein EntA and the Fe-S cluster protein NfuA are also required for virulence in the *G. mellonella* infection model (Penwell et al., 2012; Zimbler et al., 2012). Finally, TonB_1_ and TonB_2_ together but not individually are required for full virulence of *A. baumannii* ATCC 19606^T^ in the *G. mellonella* infection model (Zimbler et al., 2013). It has been reported that clinical *A. baumannii* strains display an enhanced ability to resist iron starvation when compared to a non-human isolate (Antunes et al., 2011). Together these results support the importance of iron homeostasis to the success of *A. baumannii* in the host. A summary of identified and putative iron homeostasis systems in *A. baumannii* is depicted in Figure 1A.

## Non-iron metals and *A. baumannii*

Non-iron metal homeostasis in *A. baumannii* is less well-understood; however, systems for zinc uptake and utilization have been investigated. *A. baumannii* encodes a conserved inner membrane ABC zinc transporter called ZnuABC. ZnuABC systems have been identified in numerous bacterial species such as *Campylobacter jejuni, Salmonella, Neisseria meningitidis, E. coli*, and *Y. pestis* and in some cases demonstrated to be required for virulence (Patzer and Hantke, 1998; Campoy et al., 2002; Ammendola et al., 2007; Davis et al., 2009; Stork et al., 2010)*.* The *A. baumannii* ZnuABC transporter is up-regulated when *A. baumannii* is starved for zinc and in murine lungs during *A. baumannii* pneumonia (Hood et al., 2012). ZnuB, the inner membrane permease, is required for growth in zinc-limiting conditions and for bacterial growth in the mouse pneumonia model (Hood et al., 2012). *A. baumannii* also encodes the Fur family zinc uptake regulator Zur, a zinc-sensing repressor that recognizes a conserved Zur box DNA sequence upstream of target genes when zinc-bound. A putative list of Zur target genes has been identified and include candidate outer membrane zinc transporters ZnuD_1_ and ZnuD_2_, a TonB/ExbB/ExbD system, and genes involved in intracellular zinc homeostasis (Hood et al., 2012). These findings are consistent with Zur regulons in other bacteria such as *M. tuberculosis, N. meningitidis, Corynebacterium glutamicum*, and *Y. pestis* (Maciag et al., 2007; Li et al., 2009; Schroder et al., 2010; Pawlik et al., 2012). Furthermore, the expression of *znuA, znuB, znuC, znuD*_*1*_, *znuD*_*2*_, and *tonB* increases when zinc is limiting. The putative outer membrane transporters ZnuD_1_ and ZnuD_2_ have homology to the ZnuD described in *N. meningitidis*, which was demonstrated to be involved in both zinc and heme acquisition (Stork et al., 2010; Kumar et al., 2012)*.* Although TonB-dependent receptors have been shown to be involved in zinc acquisition, no TonB system has yet been demonstrated to be directly involved in zinc acquisition through the transfer of energy to TonB-dependent receptors. Additional work is required to clarify functions for these proteins and their role in the varying *in vivo* niches of *A. baumannii.* Finally, further studies are needed to identify and characterize bacterial systems for import and utilization of other non-iron metals. For example, studies investigating the role of the host protein CP in the sequestration of zinc and manganese from bacterial pathogens demonstrate a requirement of manganese for *A. baumannii* growth (Damo et al., 2013). In *S. aureus*, CP-mediated manganese sequestration inhibits manganese-dependent superoxide defenses, and it is possible that similar inhibition is occurring in *A. baumannii* (Kehl-Fie et al., 2011). A summary of identified and putative zinc homeostasis systems within *A. baumannii* is depicted in Figure 1B.

## Adherence of *A. baumannii* to host cells

During infection, *A. baumannii* colonizes several niches and therefore interacts with and likely adheres to numerous cell types. *A. baumannii* has been shown to adhere to various cell types including human lung, laryngeal, nasopharyngeal, and cervical epithelial cells, *Candida albicans* filaments, and *Acanthamoeba castellanii* amoebal cells (Lee et al., 2006; Choi et al., 2008; Gaddy et al., 2009; Eijkelkamp et al., 2011b; Tamang et al., 2011). Furthermore, different strains of *A. baumannii* display varying capacities for cell adherence, and not all strains encode the same attachment machinery (Lee et al., 2006, 2008; de Breij et al., 2010; Eijkelkamp et al., 2011b). *A. baumannii* associates with epithelial cells by means of fimbria on the bacterial cell surface; however, additional bacterial proteins have been implicated in host epithelial cell attachment (Lee et al., 2006, 2008). *A. baumannii* outer membrane protein A (OmpA) is required for adherence to *C. albicans* filaments and epithelial cells (Choi et al., 2008; Gaddy et al., 2009). Notably, OmpA is enriched in bacterial membranes when iron is replete (Nwugo et al., 2011). Thus, it is possible that fluctuations in iron availability within host niches regulate the expression of OmpA, and possibly other OMPs, which promotes binding to host cells within a diverse array of tissue types. Furthermore, the ability of OmpA to mediate host cell binding is due at least in part to interactions with fibronectin (Smani et al., 2012b). Fibronectin also interacts with *A. baumannii* EF-Tu, Omp33, an unnamed TonB-dependent copper receptor, and a 34 kDa outer membrane protein (Gaddy et al., 2009; Dallo et al., 2012; Smani et al., 2012b, 2013). Interestingly, TonB_2_ a protein important for *A. baumannii* iron homeostasis, is also required for *A. baumannii* binding to fibronectin and epithelial cells (Zimbler et al., 2013). Beyond fibronectin, the autotransporter protein Ata mediates adhesion to Type IV collagen, and outer membrane protein ChoP interacts with the host protein platelet activating factor receptor (PFAR) (Bentancor et al., 2012; Smani et al., 2012a). It is possible that iron availability regulates outer membrane protein levels, such as OmpA, to levels that would promote binding to host cells. Overall, the number of factors interacting with host cells suggests that *A. baumannii* employs a multi-faceted strategy to host cell adherence that likely facilitates its capacity to thrive in a diverse array of tissue types.

## *A. baumannii* invasion of host cells

Following attachment, *A. baumannii* can invade host cells. Susceptible cell types include human lung, laryngeal, and cervical epithelial cells, and *A. castellanii* cells, yet sensitivity to invasion varies by cell type (Choi et al., 2008; Gaddy et al., 2009; Tamang et al., 2011). Likewise, different strains of *A. baumannii* have varying invasive potential (Choi et al., 2008). *A. baumannii* enters epithelial cells by way of a microfilament- and microtubule-dependent, zipper-like mechanism and upon internalization, localizes to membrane-bound vacuoles (Choi et al., 2008). Clathrin and β-arrestins are also engaged during the uptake of *A. baumannii* into lung epithelial cells (Smani et al., 2012a). *A. baumannii* can then persist within host cells; however, no intracellular replication has been reported. Bacterial proteins that are required for invasion and intracellular persistence include OmpA, Omp33, blue-light-sensing A (BlsA), and phospholipase D (Choi et al., 2008; Gaddy et al., 2009; Jacobs et al., 2010; Mussi et al., 2010; Smani et al., 2013). Several iron-related genes are also required for intracellular survival. Acinetobactin synthesis and uptake proteins BasD and BauA are expressed by intracellular *A. baumannii* and are required for survival within epithelial cells (Gaddy et al., 2012). Likewise, EntA and NfuA are both necessary for *A. baumannii* intracellular persistence (Penwell et al., 2012; Zimbler et al., 2012). The requirement for NfuA may be due to its role in the alleviation of oxidative damage or indirectly through the formation of Fe-S clusters on NfuA targets that are required for intracellular persistence (Zimbler et al., 2012). Together these results demonstrate the importance of iron homeostasis for *A. baumannii* intracellular survival and support the idea that the intracellular compartment is an iron-starved environment.

## Physiological relevance of *A. baumannii* intracellular persistence

**Figure 2:**
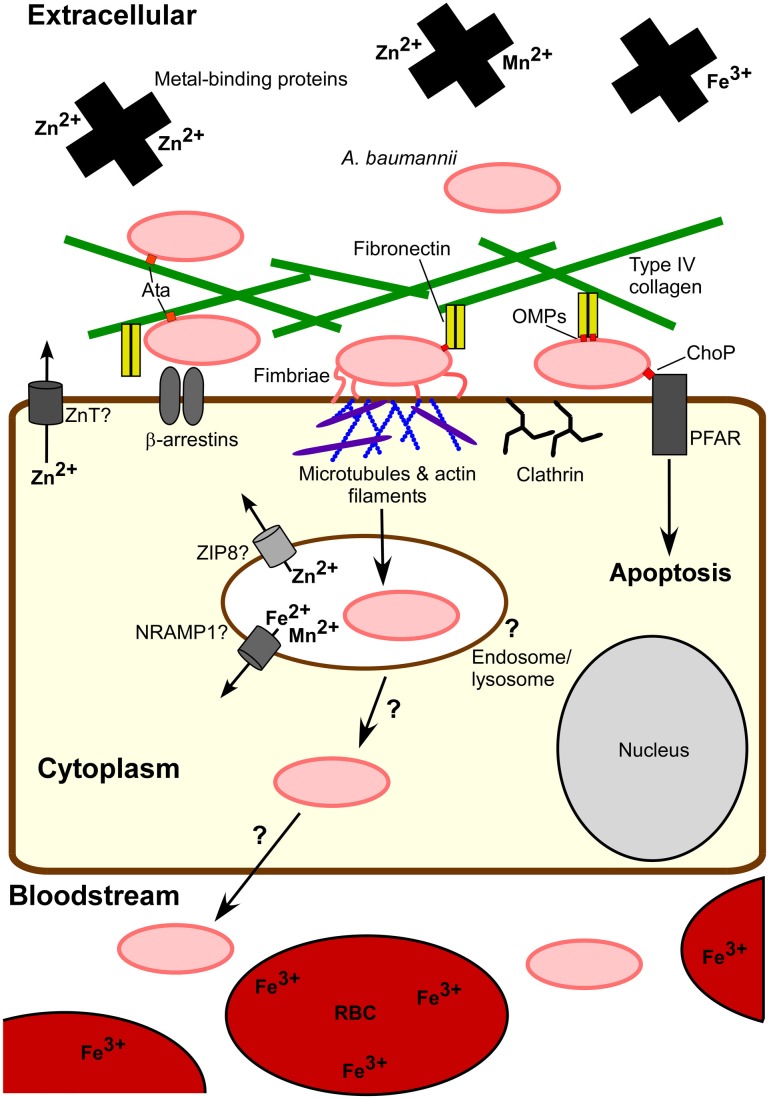
***A. baumannii-*host cell interactions**. Extracellular *A. baumannii* experiences a metal-limited environment at least in part due to metal-binding host proteins and intracellular localization of metals, e.g., red blood cells (RBCs). *A. baumannii* attaches to host cells through interactions between fibronectin and bacterial outer membrane proteins (OMPs), such as OmpA, as well as Type IV collagen via Ata. These OMPs may bind to other host cell proteins, for example the interaction of OMP ChoP with platelet activating factor receptor (PFAR). Fimbriae also contribute to eukaryotic cell adherence. Invasion into epithelial cells occurs via host cell actin filament and microtubule reorganization, recruitment of β-arrestin and clathrin, and a bacterial uptake via a zipper-like mechanism. Although *A. baumannii* can persist intracellularly and includes entry into endosomes, the intracellular trafficking of *A. baumannii* is not clear. Eukaryotic cell proteins NRAMP1, ZIP8, ZnTs, and other transporters pump essential metals out of endocytic vesicles and the cytoplasm, limiting the intracellular pool available to pathogens. The physiological relevance of the intracellular lifecycle of *A. baumannii* remains to be understood. One consequence of host cell exposure to *A. baumannii* is apoptosis. It is possible that cell death and/or host cell invasion serve to permit *A. baumannii* dissemination to deeper tissues leading to invasive disease.

A better understanding of both bacterial and host factors involved in invasion and intracellular persistence will help to elucidate the role of these processes during infection. The fact that *A. baumannii* can be killed by macrophages and other phagocytic cells, and the lack of evidence for intracellular replication or long term intracellular survival direct us away from the designation of *A. baumannii* as a predominately intracellular pathogen. Nonetheless, the ability to invade and persist within eukaryotic cells advocates that *A. baumannii*'s intracellular phase is physiologically relevant. First, *A. baumannii* entry into host cells may promote invasive disease. Although *A. baumannii* can survive within host cells, contact with cells triggers an apoptotic cell death, which is mediated at least in part by PARP and the iron-regulated protein OmpA (Choi et al., 2005; Gaddy et al., 2009; Smani et al., 2012a). Apoptosis may facilitate *A. baumannii* passage through the cell layer by eliminating the cellular barrier. One possibility is that intracellular passage may provide access to the basal side of cells through which *A. baumannii* can transit across to the underlying tissue. Additionally, *A. baumannii* stimulates a robust innate immune response; thus, relocating within host cells may afford *A. baumannii* a means to evade host immune attack. Figure 2 illustrates known aspects of *A. baumannii-*host cell interactions and highlights current questions regarding the intracellular lifecycle of *A. baumannii* with particular focus on the interplay between host and bacterial metal homeostatic mechanisms.

## Conclusions

Many vertebrates sequester nutrient metals as a defense mechanism against invading pathogens through the use of several transport and storage proteins. In order to obtain metals in the face of host metal-limiting strategies *A. baumannii* employs specialized systems that are required for growth in numerous models of *A. baumannii* infection. Future work is required to define the specific bacterial strategies for maintenance of metal homeostasis in the different host niches that *A. baumannii* occupies. During *A. baumannii* infection, the host rapidly deploys phagocytic cells and other effectors to clear infection. While *A. baumannii* has not been observed to persist within these phagocytic cells, *A. baumannii* does bind to fibronectin and epithelial cells, permitting bacterial invasion and intracellular persistence. A predominately intracellular lifecycle for *A. baumannii* is not supported by current studies, but future research will clarify the role of *A. baumannii* host cell invasion and intracellular persistence. Importantly, elucidation of *A. baumannii* mechanisms for metal transport and maintenance and their contributions to *A. baumannii* success within the various infection sites may reveal targets for the generation of new antimicrobials against this widely antibiotic-resistant organism.

## Authors contributions

Brittany L. Mortensen reviewed the literature and contributed to writing and revising this manuscript. Eric P. Skaar contributed to writing and revising this manuscript.

### Conflict of interest statement

The authors declare that the research was conducted in the absence of any commercial or financial relationships that could be construed as a potential conflict of interest.
